# The Evaluation of (1*R*,4*R*,7*R*,10*R*)-α,α′,α″,α‴-Tetramethyl-1,4,7,10-tetraazacyclododecane-1,4,7,10-tetraacetic Acid (DOTMA) as a Chelator for Zirconium-89

**DOI:** 10.3390/molecules30204129

**Published:** 2025-10-19

**Authors:** Darpan N. Pandya, Pere Miro, Michael A. Sinnwell, George B. Crull, Thaddeus J. Wadas

**Affiliations:** 1 Department of Radiology, University of Iowa, Iowa City, IA 52242, USA; 2 Department of Chemistry, University of Iowa, Iowa City, IA 52240, USA; pere-miro@uiowa.edu (P.M.); michael-sinwell@uiowa.edu (M.A.S.); george-crull@uiowa.edu (G.B.C.); 3 MATFab Facility, University of Iowa, Iowa, IA 52242, USA

**Keywords:** tetraazamacrocycle, zirconium-89, positron emission tomography, radiochemistry

## Abstract

Recently, macrocycles such as 1,4,7,10-tetraazacyclododecane-1,4,7,10-tetraacetic acid (DOTA) have been observed to form zirconium-89 (^89^Zr: t_½_ = 78.4 h, β+: 22.8%, E_β+max_ = 901 keV; EC: 77%, E_γ_ = 909 keV)-complexes with excellent in vivo stability. In this report, we describe (*1R,4R,7R,10R*)-α,α′,α″,α‴-tetramethyl-1,4,7,10-tetraazacyclododecane-1,4,7,10-tetraacetic acid (DOTMA) as an ^89^Zr chelator. Using [^89^Zr]ZrCl_4_, [^89^Zr]Zr-DOTMA was prepared in 99% radiochemical yield and a molar activity of 1055 ± 6 MBq/µmol. In vitro studies revealed a LogP value of −2.97± 0.02 and a radiometal complex that was inert when challenged with 1000-fold excess EDTA or high concentrations of biologically relevant metal ions. Finally, biodistribution studies revealed that the radiometal complex demonstrated in vivo behavior that was like [^89^Zr]Zr-DOTA and superior to [^89^Zr]Zr-DFO. Despite these promising observations, the elevated temperature required to form the [^89^Zr]Zr-DOTMA complex and the lack of derivatives available for bioconjugation will require additional ligand engineering to improve its utility for future nuclear medicine applications.

## 1. Introduction

Zirconium-89 (^89^Zr: t_1/2_ = 78.4 h; β^+^; 23%, 909 keV) chelator development remains an active area of research, with numerous ligands being reported in the literature by a vibrant and creative research community [1,2,3,4,5,6,7,8,9,10,11,12,13,14,15,16,17,18,19,20]. While many coordinating units such as hydroxamate, hydroxyisopthalamide, terepthalamide, and hydroxypyridinone have been evaluated as part of acyclic and cyclic ligands, the most widely used chelation strategy remains focused on the use of [^89^Zr]Zr-oxalate and desferrioxamine (DFO) or its analogues. However, potential challenges exist with this strategy and include ^89^Zr transchelation in vivo. For example, Ulaner et al. used [^89^Zr]Zr-DFO-trastuzumab and clinical PET/CT to identify HER2^+^, distant metastases in women diagnosed with HER2^−^, and primary disease [21]. While using [^89^Zr]Zr-DFO-trastuzumab and clinical PET/CT demonstrated success, a false-positive lesion detection rate of 30%, with half of those observed in osseous metastases, was considered a limitation of this imaging strategy [21]. The authors suggested that this large false-positive rate may result from the ^89^Zr^4+^ ion being released from the DFO-trastuzumab conjugate and non-specifically localizing in bone at sites of metastasis. These findings demonstrate the importance of improving the stability of ^89^Zr complexes used in radiopharmaceutical applications. Furthermore, while these hydroxamate derivatives have been used in Zr-89 immuno-PET applications, the ligands have not been shown to stably chelate a variety of therapeutic radionuclides such as ^177^Lu, ^212^Pb, or ^225^Ac, which are currently being evaluated in PRRT- or radioimmunotherapy-based clinical trials [22]. Currently, this represents manufacturing and regulatory challenges for research groups seeking to translate their novel theranostic approaches since two separate bioconjugates would be required for imaging and radiotherapy. 

**Figure 1:**
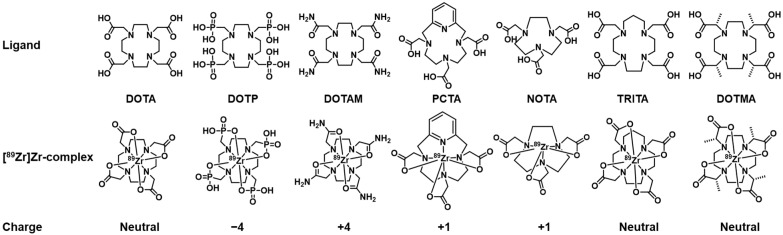
Structures of polyazamacrocycles and their ^89^Zr-complexes. Since single crystal x-ray crystallography was not performed on Zr-DOTMA, the atomic connectivity of [^89^Zr]Zr-DOTMA is a proposed structure based upon solid-state NMR analysis and DFT calculations.

Over the last decade, several research groups have sought to overcome these perceived limitations by reexamining the potential utility of azamacrocyles for this purpose. Historically, azamacrocycles have been extensively studied in molecular imaging and radiotherapy research given their ability to stably chelate a variety of radioactive and non-radioactive metals that could be utilized in radiotherapy, single photon emission computed tomography (SPECT), positron emission tomography (PET), and magnetic resonance imaging (MRI) [23,24]. Several groups have actively championed azamacrocycles as alternative ^89^Zr chelators after Holland et al. described the preparation of [^89^Zr]Zr-oxalate and [^89^Zr]ZrCl_4_ with excellent molar activity; these studies resulted in new guidelines for the long-term storage of ^89^Zr-based radiopharmaceuticals and methods for utilizing the reactivity of [^89^Zr]ZrCl_4_ in the radiochemical synthesis of ^89^Zr-labelled antibodies without loss of antigen reactivity [25,26,27]. Additionally, using [^89^Zr]ZrCl_4_ as a zirconium-89 source, Pandya et al. was able to synthesize and evaluate [^89^Zr]Zr-DOTA, [^89^Zr]Zr-DOTP, and [^89^Zr]Zr-DOTAM and their non-radioactive analogs (Figure 1). Using single crystal x-ray diffraction analysis, the molecular structure of Zr-DOTA was elucidated [10]. This study definitively demonstrated that all four macrocycle nitrogen atoms and all four acetate pendant arms participate in Zr^4+^ ion coordination to form an octa-coordinate complex. Furthermore, it provided a structure-based rationale to explain the extraordinary and unexpected stability of the resulting [^89^Zr]Zr-DOTA complex, which was superior to [^89^Zr]Zr-DFO in vitro and in vivo. In a subsequent publication, Pandya et al. described the synthesis and characterization of [^89^Zr]Zr-PCTA, [^89^Zr]Zr-TETA, [^89^Zr]Zr-TRITA, and [^89^Zr]Zr-NOTA (Figure 1) and their analogous non-radioactive complexes to determine how changes in the macrocycle’s structure affect the chemical and physical properties of the respective radiometal complex [11].

The solid-state structures of Zr-TRITA, Zr-PCTA, and Zr-NOTA were described, and these structural data reinforced what was observed with the Zr-DOTA crystal structure. Additionally, the preparation of [^89^Zr]Zr-TRITA, [^89^Zr]Zr-NOTA, and [^89^Zr]Zr-PCTA was achieved. Despite the poor in vitro and in vivo behavior of [^89^Zr]Zr-TRITA, [^89^Zr]Zr-NOTA and [^89^Zr]Zr-PCTA demonstrated robust in vitro stability and in vivo behavior, which in many aspects was superior to that of [^89^Zr]Zr-DFO. Furthermore, several groups have begun to demonstrate how ^89^Zr-azamacrocyle chemistry can be applied to clinical questions through several first-in-human studies. For example, Prive et al. compared [^89^Zr]Zr-DOTA-PSMA-617 and [^89^Zr]Zr-DOTA-PSMA-I&T with [^177^Lu]Lu-DOTA-PSMA-617 and [^177^Lu]Lu-DOTA-PSMA-I&T, respectively, in a mouse model of PSMA^+/-^ prostate cancer [28]. The ^89^Zr radiopharmaceuticals performed well with the amount of radioactivity in the blood and tumors of animals receiving either the ^89^Zr or ^177^Lu agents, as they are similar. More interestingly, [^89^Zr]Zr-DOTA-PSMA-617 was evaluated in a first-in-human study in a 64-year-old subject with suspected biochemically recurrent prostate cancer. Clinical PET imaging revealed excellent image contrast that improved over time due to the rapid excretion of the radiopharmaceutical from non-target tissues and the relatively long half-life that is intrinsic to ^89^Zr. Adverse events were not observed, suggesting that the use of this radiochemistry strategy was well-tolerated. More importantly, due to the excellent image contrast, a small focal lesion in the prostate bed, which was suspected of local recurrence, was observed at the 48 h imaging time point. A further study conducted by Rosar et al. compared the utility of [^89^Zr]Zr-PSMA-617 PET/CT with [^68^Ga]Ga-PSMA-11 PET/CT imaging in seven subjects with suspected biochemical recurrent prostate cancer [29]. In 75% of subjects, [^89^Zr]Zr-PSMA-617 PET/CT imaging revealed at least one prostate cancer lesion that was not detected by the ^68^Ga-based radiopharmaceutical. Although these reports evaluated small patient cohorts, they independently validated the ^89^Zr-tetraazamacrocycle radiochemistry paradigm and gratifyingly demonstrated the important role it may play in improving clinical care.

In this report, we extend our investigations in ^89^Zr-tetraazamacrocycle chemistry by describing the use of (*1R,4R,7R,10R*)-α,α′,α″,α‴-tetramethyl-1,4,7,10-tetraazacyclododecane-1,4,7,10-tetraacetic acid (DOTMA) (Figure 1) as an ^89^Zr chelator. We chose to investigate this additional tetraazamacrocycle to understand the influence of the methyl groups on radiochemistry and stability and to determine how closely those observations paralleled what is reported with metal–DOTMA complexes already described in the literature. Furthermore, we rationalized that if the radiochemistry and in vivo behavior of [^89^Zr]Zr-DOTMA was like that of [^89^Zr]Zr-DOTA, then the four methyl groups located on the pendant arms could be modified in subsequent studies to generate a bifunctional chelator version of DOTMA that would be valuable to the nuclear medicine community as ^89^Zr-polyazamacrocyle research expands over time.

## 2. Results and Discussion

Over the last few decades, research involving the use of ^89^Zr has accelerated, with excellent scientific contributions from a global research community [3,7,30,31,32,33,34,35,36]. In addition to optimized production and purification protocols that yield this radiometal in excellent purity and high molar activity, significant effort has been expended to develop novel ligands to ensure the stable chelation of ^89^Zr [1,2,4,5,6,8,9,10,11,12,13,14,15,16,27,31,37,38]. The stable chelation of ^89^Zr is required to inspire confidence in the ^89^Zr-radiopharmaceutical PET/CT imaging results if clinical translation is to be realized. ^89^Zr complex instability has already been identified in numerous preclinical studies and postulated as the source of a large false-positive scan rate in at least one Phase 0 clinical trial [25,26,39,40,41,42,43]. Since the reality of FDA-approved ^89^Zr-mAbs is on the horizon, with at least one Phase 3 trial (NCT 03849118) successfully meeting its clinical endpoints [44], solving the problem of ^89^Zr-chelate instability remains an active area of research. If resolved, it will improve clinical imaging and positively impact patient care on a broad scale. Additionally, the recent first-in-human studies using ^89^Zr-tetraazamacrocycle complexes as part of a broader PET imaging strategy has demonstrated that this approach is safe, yields acceptable dosimetry, and may have a larger role to play when used to determine personalized dosimetry in advance of targeted, systemic radiotherapy [28,45,46].

For nearly a decade, we have evaluated cyclen- and cyclam-based macrocycles as ^89^Zr chelators with the overall goal of developing ultra-stable radiometal chelates that could be used in the immuno-PET or theranostic paradigms [10,11]. In these studies, we observed that the macrocycles DOTA, NOTA, and PCTA formed ^89^Zr complexes with excellent in vitro and in vivo stability. In this report, we extend those studies by evaluating the ligand (*1R,4R,7R,10R*)-α,α′,α″,α‴-tetramethyl-1,4,7,10-tetraazacyclododecane-1,4,7,10-tetraacetic acid (DOTMA) as a chelator for zirconium-89. Given its extensive use in preclinical MRI contrast agent development and its structural similarity to DOTA, we theorized the resulting [^89^Zr]Zr-DOTMA complex would exhibit similar behavior to that of [^89^Zr]Zr-DOTA and yield another ultra-stable radiometal complex that can be utilized in ^89^Zr-based imaging applications [47,48]. Most importantly, the four additional methyl groups that are located on the pendant arms of the macrocycle offer an accessible derivatization point, which may be used to convert this ligand into a bifunctional chelator.

### 2.1. Structural Characterization of Zr-DOTMA

As with our previous studies and to assist in our understanding of the chemical reactivity of this ligand with ^89^Zr, we attempted to characterize the Zr-DOTMA complex. Unfortunately, the synthesis of Zr-DOTMA was problematic. Any reaction conditions attempted either revealed the presence of starting materials suggesting either that no reaction occurred or that a reaction occurred that produced a white powder, which was insoluble in every conventional solvent tested. This insolubility prevented any rigorous characterization using standard analytical techniques such as solution NMR, mass spectrometry, or single crystal X-ray diffraction analysis. Thus, to provide some characterization data, we completed solid-state NMR of Zr-DOTMA and compared its spectrum with that of the DOTMA ligand (Figures S1 and S2). Briefly, the C-13 spectrum of the DOTMA ligand is composed of four groups of resonances consistent with CH_3_, CH_2_, CH, and COO^−^. The spectra are assigned as COO^−^ near 182 ppm, CH at 58 ppm, CH_2_ centered near 48 ppm, and four methyl resonances near 9.5 ppm. The CH_3_ is attached to a chiral carbon atom at a stereogenic center. In contrast to the ligand spectra, the Zr-DOTMA spectrum is comprised of a major crystalline phase, apparently with Z’ = 1 having resonances consistent with CH_3_, CH_2_, CH, and COO at 11, 47, 66, and 179 ppm, respectively. It also has minor resonances near 52, 62, and 174 ppm. The resonances near 174 are composed of at least three unique carbons but, based on linewidth, appear to be crystalline in nature. The methyl groups are magnetically inequivalent. Importantly, no un-complexed DOTMA ligand was observed in the Zr-DOTMA C-13 spectrum, which would be consistent with the formation of the Zr-DOTMA complex.

The N-15 spectrum of the DOTMA ligand is composed of three resonances with similar chemical shifts likely suggesting disorder in the conformation, but they could also suggest multiple phases. In contrast, the N-15 spectrum of the Zr-DOTMA represents a single resonance, which is shifted by about 30 ppm. The single resonance is consistent with a single environment for the nitrogen. The nitrogen spectrum is consistent with a high-symmetry environment, and similar to the C-13 spectra, no un-complexed DOTMA ligand was observed in the Zr-DOTMA sample, which further supports the formation of the Zr-DOTMA complex.

**Figure 2:**
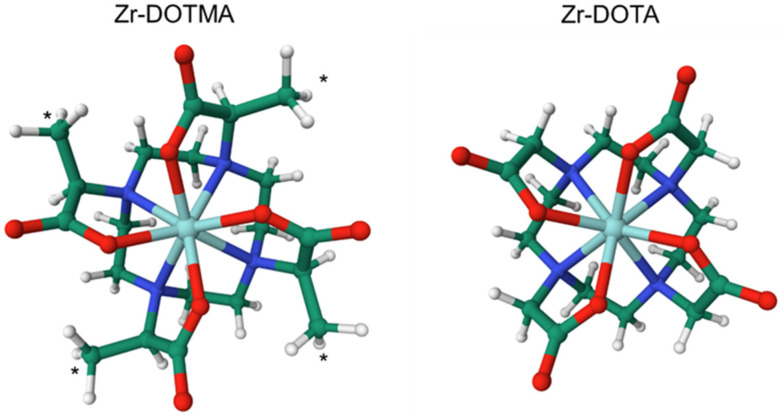
DFT-optimized structures of Zr-DOTMA and Zr-DOTA. Asterix (*) denotes the position of the additional methyl groups of the Zr-DOTMA structure. Calculations and modelling support an octa-coordinate coordination environment, which is very similar to the coordination environment of Zr-DOTA. Bond lengths and angles vary by 0.003 Å and 0.09°, respectively. Legend: gray, carbon; white, hydrogen; blue, nitrogen; red, oxygen; teal, zirconium.

To further aid our structural understanding of Zr-DOTMA, we performed DFT calculations using the Zr-DOTA crystal structure and NMR data as guides. Based upon those calculations, a model of the proposed Zr-DOTMA structure in comparison with that of Zr-DOTA is presented in Figure 2 (Table S1). The DFT-optimized structure of Zr-DOTMA is in good agreement with the experimentally elucidated crystal structure of Zr-DOTA [10], with bond and angle differences of ca. 0.038 Å and ca. 1.38°, respectively. These small differences can be attributed to differences between the crystal environment and the simulated aqueous environment (e.g., crystal packing). This supports the choice of method to characterize the structure of both Zr-DOTA and similar complexes for which a crystal structure is not available, such as Zr-DOTMA. In this case, the Zr environment is very similar to Zr-DOTA, with bond and angle differences of only ca. 0.003 Å and ca. 0.09°, respectively. These results, when considered with the NMR and elemental analysis data, provide circumstantial evidence to support the formation of the Zr-DOTMA complex.

Based upon the previously solved Zr-DOTA crystal structure and the work of Aime (Vida infra), a possible explanation can be rationalized to explain the difficulty in forming this complex [49]. In their publication, which investigated Gd-DOTMA complexes as MRI contrast agents, the research team observed that the methyl groups of the DOTMA pendant arms were positioned anti to the metal ion. This ligand configuration was believed to be responsible for negatively influencing the formation kinetics of any resulting Ln^3+^-DOTMA complex. Thus, we speculate that this same ligand conformation is responsible for the forcing conditions needed to form [^89^Zr]Zr-DOTMA and would be consistent with our previous observations regarding the formation of [^89^Zr]Zr-TETA [11]. Disappointingly, being unable to characterize Zr-DOTMA using solution NMR, mass spectrometry, or single crystal diffraction analysis remains a limitation of this work.

### 2.2. Radiochemistry

**Scheme 1:**
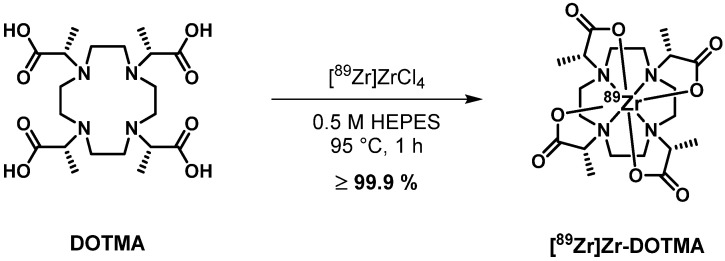
Radiochemical synthesis of [^89^Zr]Zr-DOTMA using [^89^Zr]ZrCl_4_.

Following our previously published studies, we first attempted to radiolabel DOTMA using [^89^Zr]Zr(ox)_4_ [25,50]. However, radiochemical yields were poor (Figure S3 and Table S2); we were only able to obtain a radiochemical yield of 36 ± 5%, which was consistent with results obtained when we attempted the preparation of [^89^Zr]Zr-DOTA under similar conditions [10]. Thus, we used [^89^Zr]ZrCl_4_ (Scheme 1 and Figure S4) as a radioactive precursor and a variety of reaction buffers (Table S3 and Figure S5) and reaction temperatures (Table S4 and Figure S6) to prepare [^89^Zr]Zr-DOTMA.

**Figure 3:**
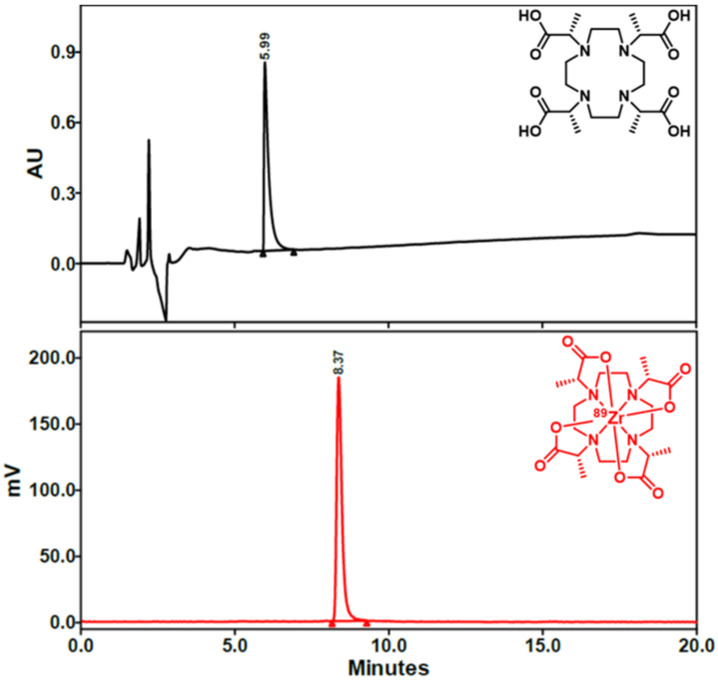
Quality control of [^89^Zr]Zr-DOTMA by radio-HPLC. UV-HPLC chromatogram (201 nm) of un-complexed ligand DOTMA (**top**) compared with radio-HPLC chromatogram of [^89^Zr]Zr-DOTMA (**bottom**). HPLC analysis revealed a peak with a retention time of 8.37 min in the radio-chromatogram. This retention time was distinct from the peak associated with the DOTMA ligand that was observed in the UV–vis chromatogram and suggested formation of the radiometal complex.

The optimized radiochemical synthesis conditions are presented in Table S5. Like [^89^Zr]Zr-DOTA, an elevated reaction temperature was required to generate [^89^Zr]Zr-DOTMA. The radiochemical yield and purity of [^89^Zr]Zr-DOTMA complex were confirmed by radio-TLC (Figure S7) and radio-HPLC (Figure 3). HPLC analysis revealed a peak with a retention time of 8.37 min in the radio-chromatogram (Figure 3). This retention time was distinct from the peak associated with the DOTMA ligand that was observed in the UV–vis chromatogram and suggested formation of the radiometal complex. The molar activity (A_m_) for [^89^Zr]Zr-DOTMA was found to be 1055 ± 6 MBq/µmol and was comparable with other A_m_ values associated with ^89^Zr-complexes previously reported in the literature [10,11]. When we consider the ^89^Zr-azamacrocycles evaluated to date, the ease of radiochemical synthesis can be qualitatively ordered as [^89^Zr]Zr-PCTA > [^89^Zr]Zr-NOTA > [^89^Zr]Zr-DOTA >> [^89^Zr]Zr-DOTMA [10,11].

### 2.3. Lipophilicity (LogP) and In Vitro Stability Studies

Lipophilicity (LogP) represents a fundamental physiochemical property that influences the in vivo performance of a radiopharmaceutical [51]. While LogD_7.2_ may also be used, we chose LogP to be consistent with our previously reported results. Based upon the water/octanol partition method, [^89^Zr]Zr-DOTMA demonstrates hydrophilic character and suggests that renal excretion would be a preferred route of elimination after in vivo injection [10,11]. Interestingly, [^89^Zr]Zr-DOTMA demonstrated a LogP value (−2.97 ± 0.02; Table S6) that was more negative than the LogP value of [^89^Zr]Zr-DFO (−2.83 ± 0.03) but more positive than the LogP of [^89^Zr]Zr-PCTA (−3.09 ± 0.03), which probably reflects a combination of the pyridyl ring and the radiometal complex’s expected 1^+^ charge. However, its LogP was more positive than the value reported for [^89^Zr]Zr-DOTA (−3.80 ± 0.04), and this may be attributed to the influence of the four extra methyl groups on the pendant arms of the DOTMA macrocycle [10].

The in vitro stability of [^89^Zr]Zr-DOTMA was evaluated by challenging it with excess EDTA, high concentrations of biologically relevant metal ions, or human serum proteins over a seven-day time course [10,11]. These experiments revealed that [^89^Zr]Zr-DOTMA was stable to a 1000-fold excess EDTA challenge (at pH 5 or pH 7; Table 1).

**Figure 4:**
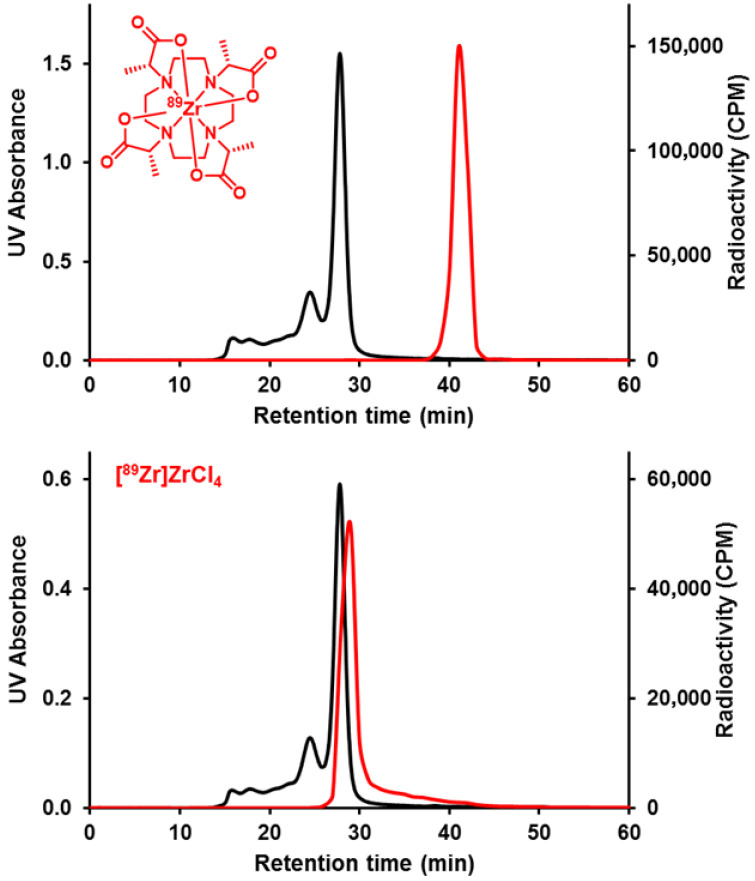
In vitro serum stability by HPLC. UV-SE-HPLC (220 nm, black) and radio-SE-HPLC chromatogram (red) of [^89^Zr]Zr-DOTMA (**top**) and [^89^Zr]ZrCl_4_ (**bottom**) in serum after 7 days. Black lines are the UV absorbance due to the human serum components, and red lines are the radiotracer associated with ^89^Zr complexes. The radiometal complex elutes at approximately 39 min. This retention time is distinct from the retention times associated with serum proteins or [^89^Zr]ZrCl_4_ and suggests that the radiometal complex is inert to transchelation by serum proteins.

Additionally, no demetallation of [^89^Zr]Zr-DOTMA was observed when challenged with high concentrations of Fe^3+^, Co^2+^, Zn^2+^, Cu^2+^, Mg^2+^, Ga^3+^, or Gd ^3+^ ions. (Table 2). Finally, incubation in human serum (Figure 4, Figures S8 and S9, and Table 3) revealed that any ^89^Zr, which was added to the serum as [^89^Zr]Zr-DOTMA, was not associated with the serum proteins, suggesting that the former radiometal complex was like [^89^Zr]Zr-DOTA in its ability to resist transchelation by serum proteins.

### 2.4. Biodistribution Studies in Non-Tumor-Bearing, Normal Mice

**Figure 5:**
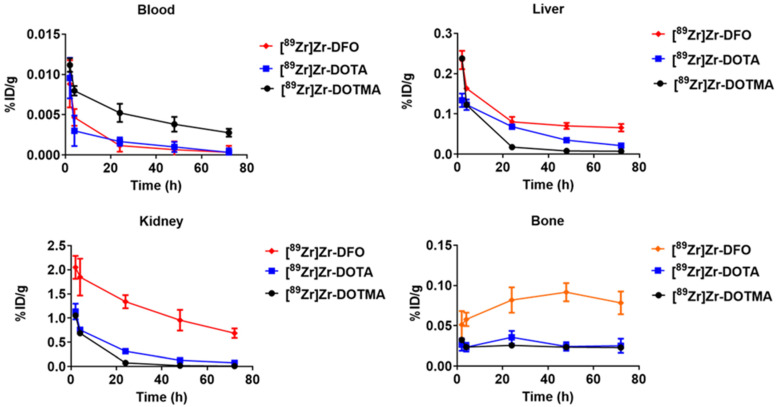
Biodistribution comparison of [^89^Zr]Zr-DOTMA with [^89^Zr]Zr-DOTA and [^89^Zr]Zr-DFO in select tissues. While retention in the blood pool was slightly higher than that of [^89^Zr]Zr-DOTA and [^89^Zr]Zr-DFO, the excretion profile of [^89^Zr]Zr-DOTMA from the liver, kidney, and bone were better than the excretion profiles of [^89^Zr]Zr-DFO and similar to [^89^Zr]Zr-DOTA. These data suggest that [^89^Zr]Zr-DOTMA may be ultra-stable in vivo and, like [^89^Zr]Zr-DOTA, may be useful in the radiolabeling of peptides and antibodies with zirconium-89. The complete biodistribution of [^89^Zr]Zr-DOTMA is depicted in Table S7 of the Supplementary Materials. [^89^Zr]Zr-DOTA and [^89^Zr]Zr-DFO data are reproduced from ref. [10].

We then evaluated the in vivo behavior of [^89^Zr]Zr-DOTMA in acute biodistribution studies involving normal mice. Results are shown in Table S7 and Figure 5. At 2 h post-injection (p.i.), animals injected with [^89^Zr]Zr-DOTMA demonstrated a slightly elevated level of blood-associated radioactivity, which decreased over the time course of the experiment. The only structural differences between [^89^Zr]Zr-DOTMA and [^89^Zr]Zr-DOTA are the four extra methyl groups, which are believed to contribute to the increased lipophilicity of the former radiometal complex. It is believed that this more positive LogP value increases the interaction between [^89^Zr]Zr-DOTMA and serum proteins once in the systemic circulation, causing this retention in the blood pool. This is not uncommon, as lipophilicity has been observed to influence a molecule’s interaction with human serum proteins [52,53]. In all other aspects, [^89^Zr]Zr-DOTMA demonstrated a comparable biodistribution profile to [^89^Zr]Zr-DOTA and was observed to be superior to [^89^Zr]Zr-DFO [10,11]. For example, at 72 h p.i., animals injected with [^89^Zr]Zr-DOTMA had 9-fold less radioactivity associated with liver tissue when compared to animals receiving [^89^Zr]Zr-DFO ([^89^Zr]Zr-DOTMA vs. [^89^Zr]Zr-DFO (liver (72 h p.i., %ID/g ± SD); *p*-value: 0.007 ± 0.001 vs. 0.066 ± 0.009; *p* < 0.0001). Similarly, radioactivity associated with kidney tissues was significantly reduced. Animals receiving [^89^Zr]Zr-DOTMA retained 69-fold less radioactivity in the kidney than did animals receiving [^89^Zr]Zr-DFO ([^89^Zr]Zr-DOTMA vs. [^89^Zr]Zr-DFO (kidney (72 h p.i., %ID/g ± SD); *p*-value: 0.010 ± 0.002 vs. 0.69 ± 0.09; *p* < 0.0001). Finally, radioactivity in the bone tissue of animals receiving [^89^Zr]Zr-DOTMA was 3-fold less than in animals injected with [^89^Zr]Zr-DFO ([^89^Zr]Zr-DOTMA vs. [^89^Zr]Zr-DFO (bone (72 h p.i., %ID/g ± SD); *p*-value: 0.023 ± 0.003 vs. 0.079 ± 0.006; *p* < 0.0001). Despite the excellent biodistribution profile, we did not perform metabolism studies since they were beyond the scope of this initial investigation. Thus, the nature of the radioactive species within the tissues remains unknown. Furthermore, we acknowledge that the observed stability of the radiometal–chelate complex may differ when incorporated into a bioconjugate. Considering that BFC derivatives of DOTMA are not commercially available, these differences are difficult to assess currently.

### 2.5. Limitations of the Current Research and Future Research Directions

This manuscript augments what is known about the ^89^Zr radiochemistry of azamacrocycles by studying the in vitro and in vivo stability of [^89^Zr]Zr-DOTMA. While it is beyond the scope of this text to provide a side-by-side comparison with every ^89^Zr chelator reported in the literature, the following points are proffered to put our results in context with the wider literature. Foremost, elevated temperatures were required to form [^89^Zr]Zr-DOTMA; this characteristic will never supplant the facile radiochemistry exhibited by numerous chelators containing hydroxamate coordinating units including DFO and DFO*. Once formed, however, [^89^Zr]Zr-DOTMA exhibits behavior that is consistent with other ^89^Zr radiometal complexes that have been reported to demonstrate excellent radiochemical stability. Within the context of azamacrocycles, [^89^Zr]Zr-DOTMA exhibits behavior that is consistent with the behavior of the ^89^Zr complexes formed with DOTA, PCTA, and NOTA. When compared specifically to DOTA, the radiochemistry to form both complexes was similar, and both radiometal chelates were of comparable inertness when challenged with exogenous ligands, metal ions, or serum proteins. The only observable differences were in the excretion profiles of individual tissues, but on aggregate, the data do not suggest one radiometal complex is superior to the other [10]. However, DOTMA does differ from DOTA in that, at present, bifunctional chelators of it are not commercially available. While DOTMA BFC derivatives have been prepared for use in MRI contrast agent development, their use has not been evaluated in nuclear medicine applications. Given these challenges, our current efforts are refocused on creating a BFC derivative of DOTMA, which could be used with a variety popular bioconjugation strategies and would be more valuable to the research community [54,55,56,57,58,59,60,61,62].

## 3. Materials and Methods

### 3.1. Reagents and Equipment

Zirconium-89 (^89^Zr: (t_1/2_ = 78.4 h, β^+^:22.8%, E_β+max_ = 901 keV; EC: 77%, E_γ_ = 909 keV) was purchased from Washington University School of Medicine (St. Louis, MO, USA) as [^89^Zr]Zr(ox)_4_ in 0.1 M oxalic acid. Unless otherwise noted, all other chemicals were purchased from MilliporeSigma (St. Louis, MO, USA), and solutions were prepared using ultrapure water (resistivity = 18 MΩ cm^−1^). The macrocyclic ligand (*1R,4R,7R,10R*)-α,α′,α″,α‴-tetramethyl-1,4,7,10-tetraazacyclododecane-1,4,7,10-tetraacetic acid (DOTMA, M.Wt. = 460.5 g/mol) was purchased from CheMatech, Inc. (Dijon, France). Radiochemistry reaction progress and purity were analyzed using an analytical HPLC system (Waters, Milford, MA, USA), which runs Empower v.3.9.0 software and is configured with a Model 1525 binary pump, Model 2707 autosampler, Model 2998 photodiode array detector, Model 1500 column heater, fraction collector, HYPERCARB C18 column (5 μm, 4.6 × 100 mm, ThermoScientific, Waltham, MA, USA), and a Model 105-s radioactivity detector (Carrol Ramsey, Berkeley, CA, USA). The DOTMA ligand and subsequent radiolabeled products were monitored at 201 nm, using a mobile phase consisting of 0.1% TFA/H_2_O (solvent A) and 0.1% TFA/acetonitrile (solvent B) and a gradient consisting of 0% B to 70% B in 20 min at a flow rate of 1.0 mL/min. In addition, radio-TLC was conducted on a Bioscan AR 2000 radio-TLC scanner equipped with a 10% methane/argon gas supply and a PC interface running Winscan v.3 analysis software (Eckert & Ziegler, Berlin, Germany). Varian ITLC-SA strips (Agilent Technologies, Santa Clara, CA, USA) and Merck C-18 TLC plates (MilliporeSigma, St. Louis, MO, USA) were employed using 50 mM EDTA (pH 5) or 1:1.0 MeOH:10% NH_4_Cl solution as eluents, respectively. Both [^89^Zr]Zr(ox)_4_ and [^89^Zr]ZrCl_4_ were used as standard controls. Radioactive samples were counted using either a CRC-25R radioisotope calibrator (Capintec, Inc., Florham Park, NJ, USA) or a PerkinElmer 2480 Wizard gamma counter (Revvity, Inc., Waltham, MA, USA) with an energy window of 500−1500 keV. Solid-state NMR spectroscopy was performed using a Bruker AVANCEIII 500 MHZ NMR spectrometer running Topspin 3.0 software (Bruker Biospin, Billerica, MA, USA). Elemental analysis was performed using an Exeter Analytical Combustion CE440 Elemental Analysis analyzer (Exeter Analytical, Inc., North Chelmsford, MA, USA).

### 3.2. Solid-State NMR Data Acquisition Parameters

NMR measurements were performed on a Bruker Avance 500 MHz spectrometer equipped with a double resonance 4 mm MASprobe (Bruker Biospin, Billerica, MA, USA) operating at a proton frequency of 500.13 MHz. All the measurements were carried out with the sample oriented at the magic angle in 4 mm zirconia rotors and under controlled temperature. The temperature was maintained at 298 K via a stream of air. MAS spectra were recorded using shaped cross-polarization pulses (ramped from 50 to 100% on the H-1 channel) at a MAS frequency of 10 kHz. The same filled rotor was used for both C-13 and N-15 measurements.

For carbon (C-13) observation, the following parameters were used: Proton 90° pulse of 4.6 μs, contact time of 2 ms, a spectral width of 300 ppm, and a relaxation delay of 5 times the proton t_1_ (ranging from 6 to 10 s). Typically, 4096 scans of 3 K data points were coadded before zero filling to 8 K and processing with an exponential weight of 25 Hz. Composite pulse decoupling using the TPPM scheme was applied on the proton channel. The carbon (C-13) spectra were refenced to TMS using external 3-Methylglutaric acid [63].

For nitrogen (N-15) observation, the following parameters were used: Proton 90° pulse of 4.25 μs, contact time of 3 ms, a spectral width of 200 ppm or 600 ppm, and a relaxation of 21 s. Typically, 1024 to 8192 scans of 4 K data points were coadded before zero filling to 8 K and processing with an exponential weight of 14 Hz. Composite pulse decoupling using the Spinal64 scheme was applied on the proton channel. The nitrogen (N-15) spectra were refenced to NH_3_ = 0 ppm using external NH_4_NO_3_ [64].

### 3.3. Synthesis of Zirconium (1R,4R,7R,10R)-α,α′,α″,α‴-Tetramethyl-1,4,7,10-tetraazacyclododecane-1,4,7,10-tetraacetic Acid (Zr-DOTMA)

ZrAcAc (702 mg, 1.44 mmol) was added to a solution of DOTMA (529 mg, 1.31 mmol) in 40 mL of methanol. The resulting solution was refluxed for 3 h. As the reaction proceeded, a white precipitate formed. It was filtered, washed with MeOH (2 × 10 mL), and dried in an oven (604 mg, 94% yield). ^13^C-NMR (500 MHz, solid state): δ 11 (m, CH_3_), 47 (m, CH_2_), 66 (m, CH), 179 (s, COO^−^) ^14^N-NMR (500 MHz, solid state): δ 40 (s, N). Elemental analysis (calculated): C, 43.86%; H, 5.89%; N, 10.23%. Elemental analysis (observed, *n* = 4): C, 43.71 ± 0.19%; H, 6.05 ± 0.04%; N, 9.27 ± 0.16%.

### 3.4. Density Functional Theory (DFT) Calculations and Modelling of the Zr-DOTMA Structure

Density functional theory (DFT) calculations were performed using the Amsterdam Density Functional package with the PBE exchange-correlation functional in conjunction with an all-electron triple-ζ Slater-type basis set with two additional polarization functions. The relativistic corrections were taken into account using the scalar relativistic zero-order regular approximation (ZORA). Dispersion effects were included using the D3 Grimme correction. Geometry optimizations were performed using the COSMO solvation model with water parameters. The nature of all stationary points and transition states were verified by the calculation of analytical vibrational frequencies. The optimized structure was visualized using Mol* 3D Viewer, which is free and available on the RCSB Protein Data Bank website: https://www.rcsb.org/ (accessed on 15 October 2025).

### 3.5. Radiochemical Synthesis of [^89^Zr]Zr-DOTMA with [^89^Zr]Zr(ox)_4_ [10]

The complexation of ^89^Zr with DOTMA was achieved by reacting 10–50 µg (0.022–0.109 µmol, 1–5 µL, 10 mg/mL in water) of DOTMA with an aliquot of [^89^Zr]Zr(ox)_4_ (0.49–0.55 mCi, 18.2–20.5 MBq) diluted in 100 µL of water and pH adjusted to 6.9–7.2 using 1.0 M Na_2_CO_3_ or 0.5 M HEPES (pH 7.0–7.2). Reactions were incubated at 99 °C for 2 h in a thermomixer (700 rpm). The formation of [^89^Zr]Zr-DOTMA complex was monitored by radio-TLC, using Varian ITLC-SA strips and 50 mM EDTA (pH 5) as the mobile phase.

### 3.6. Preparation of [^89^Zr]Zr-Chloride

[^89^Zr]ZrCl_4_ was produced using a procedure modified from the literature [26,27]. Briefly, a [^89^Zr]Zr-oxalate solution in 0.1 M oxalic acid was loaded onto an activated Waters Sep-pak Light Accell plus QMA strong anion exchange cartridge (300 Å pore size, 37–55 μm particle size, 230 μeq/g ion exchange capacity) that was prewashed with 6 mL MeCN, 10 mL 0.9% saline, and 10 mL water. The cartridge was then washed with water (>50 mL) to remove oxalic acid and the radioactivity eluted with 100% recovery of ^89^Zr by chloride ion exchange with 400–500 μL of 1.0 M HCl (aq.).

### 3.7. Radiochemical Synthesis of [^89^Zr]Zr-DOTMA with [^89^Zr]ZrCl_4_ [10]

Complexation of ^89^Zr with DOTMA was achieved by reacting 15–20 μg (0.033–0.043 µmol,15–20 μL, 1.0 mg/mL in water) of the ligand with an aliquot of [^89^Zr]ZrCl_4_ (1.1–1.6 mCi, 40.7–59.2 MBq) diluted in 200 μL of 0.5 M HEPES (pH 7.2), followed by 60 min of incubation at 95 °C in a thermomixer (600 rpm). The formation of [^89^Zr]Zr-DOTMA complex was monitored by radio-TLC, using a mobile phase consisting of 1:1 MeOH:10% NH_4_Cl on C-18 plates and 50 mM EDTA (pH 5) on Varian ITLC-SA strips. In the C-18 system, un-chelated ^89^Zr remained at the origin (R_f_ = 0), while the [^89^Zr]Zr-DOTMA complex eluted near the solvent front (R_f_ = 0.75–0.80). In the ITLC-SA system, free ^89^Zr formed a complex with EDTA and eluted with the solvent front (R_f_ ≈ 1), while the [^89^Zr]Zr-DOTMA complex elutes from origin (R_f_ = 0.10–0.15). The identity of the radioactive complex [^89^Zr]Zr-DOTMA was further confirmed by comparing its radio-HPLC elution profile to the UV-HPLC spectrum of the un-complexed ligand.

### 3.8. Determination of Partition Coefficient (LogP) [10]

The partition coefficient (LogP) was determined by adding 5 μL of [^89^Zr]Zr-DOTMA (~5 μCi; 0.19 MBq) to a mixture of 500 μL of octanol and 500 μL of water (pH 5.5). The resulting solutions (*n* = 5) were vigorously vortexed for 5 min at room temperature, then centrifuged for 5 min to ensure complete separation of layers. From each of the five sets, a 50 μL aliquot was removed from each phase into new tubes and counted separately in a gamma counter. Each organic phase was washed with water to remove any radioactivity remaining in the organic phase before gamma counting. The partition coefficient was calculated as a ratio of counts in the octanol fraction to counts in the water fraction. The LogP values were reported as an average of five measurements.

### 3.9. In Vitro EDTA Challenge Study [10]

The in vitro EDTA challenge study was performed by adding 20 μL of [^89^Zr]Zr-DOTMA complex (121–130 μCi, 4.5–4.8 MBq) to 500 μL of EDTA (10 mM, 50 mM, and 100 mM: pH 5 or pH 7) with a 1:100, 1:500, and 1:1000 ratio of radiometal complex/EDTA. The solutions (*n* = 3) were incubated at 37 °C for 7 days in a thermomixer. Samples were analyzed at 0, 1, 3, 5, and 7 days post administration to EDTA using radio-TLC, Varian ITLC-SA strips, 50 mM EDTA (pH 5) as the mobile phase, and gamma counting. All studies were performed in triplicate.

### 3.10. In Vitro Metal Competition Study [10]

To separate solutions of metal cations (iron(III) chloride, cobalt(II) chloride, zinc(II) chloride, copper(II) chloride, magnesium(II) chloride, gallium(III) nitrate, and gadolinium(III) chloride) (1.0 mM, 200 μL), [^89^Zr]Zr-DOTMA complex (0.1 mM, 20 μL, 110−119 μCi, 4.1–4.4 MBq) in PBS (pH 7.4) was added. The resulting solutions (*n* = 3) were incubated at 37 °C for 7 days in a thermomixer. Dissociation of ^89^Zr from [^89^Zr]Zr-DOTMA complex was monitored by radio-TLC at 0, 1, 3, 5, and 7 days using radio-TLC, Varian ITLC-SA strips, 50 mM EDTA (pH 5) as the mobile phase, and gamma counting. All studies were performed in triplicate.

### 3.11. In Vitro Serum Stability Study [10]

The in vitro serum stability was determined by adding 20 μL of [^89^Zr]Zr-DOTMA (113–124 μCi, 4.2–4.6 MBq) to 500 μL of human serum. The solutions (*n* = 3) were incubated at 37 °C for 7 days and were analyzed daily for 1 week by radio-TLC using Varian ITLC-SA strips, 0.1 M EDTA (pH 5) as the mobile phase, and gamma counting. Samples were also analyzed at 1, 3, 5, and 7 days via size exclusion chromatography (SEC) using a Superdex 200 10/300 GL column (GE Healthcare Life Sciences, Piscataway, NJ, USA) and phosphate buffered saline (PBS) as an eluent with a flow rate of 0.5 mL/min. Fractions (0.5 mL per tube) were collected, and the activity in each fraction was measured in a gamma counter. The percent intact radiopharmaceutical was determined by subtracting the total integrated area under the product peak from the total integrated area generated for all peaks in the chromatogram and multiplying by a factor of 100%.

### 3.12. Animal Care and Use

All animal studies were carried out in strict accordance with the recommendations in the Guide for the Care and Use of Animals of the National Institutes of Health and approved by the University of Iowa’s Institutional Animal Care and Use Committee (Protocol # 302265-001). Furthermore, all animal experiments comply with the ARRIVE guidelines and were conducted in accordance with the U.K. Animals (Scientific Procedures) Act of 1986 and associated guidelines, EU Directive 2010/63/EU for animal experiments, or the National Research Council’s Guide for the Care and Use of Laboratory Animals. All staff underwent animal handling training through the University of Iowa’s Office of Animal Research prior to the start of any experiments.

The studies described herein were designed to study the biodistribution and excretion patterns of a radiometal chelate in normal animals. These were not therapy or survival studies, and the injected mass or amount of radioactivity was considered below the limit of pharmacological action as defined by the University of Iowa’s Institutional Animal Care and Use Committee (Protocol # 302265-001). Thus, euthanasia was based upon predetermined study time points and not humane endpoints. However, in order to verify the absolute necessity of animal research for this project, to prevent repeating work that had been done by others and to confirm the lack of alternative procedures, we performed multiple PubMed scientific literature searches using numerous relevant key words, alone and/or in combination, related to the subject matter. The results of these searches confirmed that the experiments proposed are novel and in no way duplicate any previous work. Additionally, these searches verified that we used the proper methodology in our animal studies in order to screen for promising agents and minimize the number of animals used. The search also uncovered the fact that the proposed animal studies are irreplaceable and must be performed in order to assess the potential utility of these radiopharmaceuticals.

Female NIH Swiss mice (6–8 weeks old) were purchased from Inotiv, Lafayette, IN, USA. All animals were acclimated to a 12 h light/dark cycle and received food and water ad libitum for 5 days before any manipulations were performed. All animals were checked by veterinary staff daily to ensure well-being and proper environment and to monitor for signs of distress. Clinical signs of distress in laboratory rodents include decreased activity, pilo-erection, un-groomed appearance, excessive licking and scratching, self-mutilation, abnormal stance, hunched appearance, rapid or shallow respiration, grunting, dilated pupils, aggressiveness towards handler, high-pitched vocalizations, change in feeding activity, and attempts to separate from group. In the event of clinical distress, animals were euthanized after consulting the on-call veterinarian. To minimize suffering during the tail vain injection, animals were kept under deep isoflurane anesthesia. At the end point of the study, animals were anesthetized with isoflurane and were euthanized by cervical dislocation, which is consistent with the recommendations of the American Veterinary Medical Association Guidelines on Euthanasia. Animals were monitored every 30 min during the experiment, but during the experiment, none of the animals died or were found ill before experimental end point.

### 3.13. Biodistribution Studies

Biodistribution studies were conducted using a modified literature procedure [10,11]. Briefly, female NIH Swiss mice (6–8 weeks old, *n* = 6/cohort; 30 animals total) were injected with [^89^Zr]Zr-DOTMA (0.55 MBq (15 μCi)/mouse) via the tail vein and sacrificed at 2, 4, 24, 48, and 72 h p.i. Organs and tissues of interest were excised, weighed, and counted on a gamma counter. The percent injected dose per gram (%ID/g) was calculated by comparison to a weighed, counted standard for each group. At the completion of this study, 30 animals were euthanized; zero animals were found dead in their cages.

### 3.14. Statistical Analysis [10]

For statistical analysis, all plots were generated using GraphPad Prism 5.0 software (GraphPad, San Diego, CA, USA). Student’s *t*-tests (two-tailed, unpaired) were performed. *p* < 0.05 was considered statistically significant. Within the text, Student’s *t*-test data are presented as mean ± SD or mean (95% confidence intervals) and *p*-value.

## 4. Conclusions

This report describes the preparation and characterization of [^89^Zr]Zr-DOTMA in vitro and in vivo. The [^89^Zr]Zr-DOTMA complex was prepared in high radiochemical yield and molar activity. In vitro, this radiometal complex displayed excellent resistance to EDTA, metal ion, and human serum challenge. Biodistribution studies in normal mice revealed a clearance profile that was superior to [^89^Zr]Zr-DFO and comparable to [^89^Zr]Zr-DOTA. Despite these encouraging data, which augment what is known about ^89^Zr-tetraazamacrocycle chemistry, the inability to adequately characterize the analogous non-radioactive complex and the lack of bifunctional chelator derivatives of DOTMA currently limit its utility as an ^89^Zr chelator at this time.

## 5. Patents

WO2017161356A1 and WO2019/147912A1.

## Figures and Tables

**Table 1:** Chronological stability study of [^89^Zr]Zr-DOTMA in EDTA (pH 5 and pH 7) maintained at 37 °C for 7 days and analyzed by radio-ITLC (*n* = 3 at each pH and time point).

Complex	EDTA	pH	% Intact of [^89^Zr]Zr-Complex (*n* = 3)				
			0 h	1 d	3 d	5 d	7 d
[^89^Zr]Zr-DOTMA	100-fold	7.0	≥99.99	≥99.99	≥99.99	≥99.99	≥99.99
		5.0	≥99.99	≥99.99	≥99.99	≥99.99	≥99.99
	500-fold	7.0	≥99.99	≥99.99	≥99.99	≥99.99	≥99.99
		5.0	≥99.99	≥99.99	≥99.99	≥99.99	≥99.99
	1000-fold	7.0	≥99.99	≥99.99	≥99.99	≥99.99	≥99.99
		5.0	≥99.99	≥99.99	≥99.99	≥99.99	≥99.99

**Table 2:** Chronological stability study of [^89^Zr]Zr-DOTMA during metal ion competition study (PBS, pH 7.4) maintained at 37 °C for 7 days and analyzed by radio-ITLC (*n* = 3 for each metal and time point).

Time Point	Complex	% Intact of [^89^Zr]Zr-Complex (*n* = 3)						
		Fe^3+^	Zn^2+^	Co^2+^	Cu^2+^	Mg^2+^	Gd^3+^	Ga^3+^
0 h	[^89^Zr]Zr-DOTMA	≥99.99	≥99.99	≥99.99	≥99.99	≥99.99	≥99.99	≥99.99
1 d		≥99.99	≥99.99	≥99.99	≥99.99	≥99.99	≥99.99	≥99.99
3 d		≥99.99	≥99.99	≥99.99	≥99.99	≥99.99	≥99.99	≥99.99
5 d		≥99.99	≥99.99	≥99.99	≥99.99	≥99.99	≥99.99	≥99.99
7 d		≥99.99	≥99.99	≥99.99	≥99.99	≥99.99	≥99.99	≥99.99

**Table 3:** Chronological in vitro serum stability study of [^89^Zr]Zr-DOTMA in human serum maintained at 37 °C for 7 days as determined by size exclusion chromatography and radio-ITLC (*n* = 3 at each time point).

Time Point	% Intact of [^89^Zr]Zr-DOTMA (*n* = 3)	
	Radio-SE-HPLC	Radio-ITLC
0 h	≥99.99	≥99.99
1 d	≥99.99	≥99.99
3 d	≥99.99	≥99.99
5 d	≥99.99	≥99.99
7 d	99.9 ± 0.1	≥99.99

## Data Availability

The datasets used and/or analyzed during the current study are available from the corresponding author on reasonable request.

## Supplementary Materials

The following supporting information can be downloaded at https://www.mdpi.com/article/10.3390/molecules30204129/s1. Solid State ^13^C-NMR of DOTMA and Zr-DOTMA (Figure S1). Solid State 15N-NMR of DOTMA and Zr-DOTMA (Figure S2). Selected geometrical parameters of Zr-DOTA and Zr-DOTMA in gas phase and in solution (Table S1). Radiochemistry protocol using [89Zr]Zr(ox)4 for [89Zr]Zr-DOTMA (Table S2, Figure S3). Preparation of [89Zr]ZrCl4 from [89Zr]Zr(ox)4 (Figure S4). Summary of optimized reaction buffers using [89Zr]ZrCl4 for [89Zr]Zr-DOTMA (Table S3, Figure S5). Summary of optimized reaction temperatures using [89Zr]ZrCl4 for [89Zr]Zr-DOTMA (Table S4, Figure S6). Quality control of [89Zr]Zr-DOTMA by radio-TLC (Figure S7). Summary of optimized radiochemistry conditions to prepare [89Zr]Zr-DOTMA with [89Zr]ZrCl4 (Table S5). Determination of partition coefficients (LogP) (Table S6). In vitro serum stability study by Radio-ITLC (Figures S8 and S9). Complete biodistribution studies of [89Zr]Zr-DOTMA (Table S7).

## Abbreviations

DFO: Deferoxamine; PCTA: 3,6,9,15-Tetraazabicyclo [9.3.1]pentadeca-1(15),11,13-triene-3,6,9-triacetic acid; NOTA: 2,2′,2″-(1,4,7-triazacyclononane-1,4,7-triyl)triacetic acid; TRITA: 2,2′,2″,2‴-(1,4,7,10-tetraazacyclo tridecane-1,4,7,10-tetrayl)tetraacetic acid; TETA: 2,2′,2″,2‴-(1,4,8,11-tetraazacyclotetradecane-1,4,8,11-tetrayl)tetraacetic acid; EDTA: Ethylenediaminetetraacetic acid; Zr(AcAc)_4_: Zirconium(IV) acetylacetonate; Zr(ox)_4_: Zirconium(IV) oxalate; TFA: Trifluoroacetic acid; MeOH: Methanol; NH_4_Cl: Ammonium chloride; NH_4_OAc: Ammonium acetate; NaOAc: Sodium acetate; TMAA: Tetramethylammonium acetate; MES: 2-(N-morpholino)ethanesulfonic acid; HEPES: 4-(2-Hydroxyethyl) piperazine-1-ethanesulfonic acid; TRIS: Tris(hydroxymethyl)aminomethane; NMR: Nuclear magnetic resonance; HMBC: Heteroatom multiple-bond correlation; HMQC: Heteronuclear multiple quantum coherence; UV-HPLC: Ultra-violet high-performance liquid chromatography; RT: Retention time; R_f_: Retention factor; ESI-HR-MS: Electrospray ionization high-resolution mass spectrometry; HS: Human serum; ITLC: Instant thin-layer chromatography; kBq: Kilobecquerel; MBq: Megabecquerel; %ID/g: Percent injected dose per gram; P.I.: Post-injection; ROI: Region of interest; CPM: Counts per minute; TLC: Thin-layer chromatography; PET: Positron emission tomography; CT: Computed tomography;^89^Zr: Zirconium-89.
