# Supplementary material for: The Evaluation of (1R,4R,7R,10R)-α,α′,α″,α‴-Tetramethyl-1,4,7,10-tetraazacyclododecane-1,4,7,10-tetraacetic Acid (DOTMA) as a Chelator for Zirconium-89
Source: Molecules. 2025 Oct 19;30(20):4129. doi: 10.3390/molecules30204129 (PMC12566511; doi:10.3390/molecules30204129)
Supplement: Supplementary file 1 [file molecules-30-04129-s001.zip › molecules-3831623-supplementary.pdf]

The evaluation of (*1R,4R,7R,10R*)-α,α′,α″,α‴-tetramethyl-1,4,7,10-tetraazacyclododecane-1,4,7,10-tetraacetic acid (DOTMA) as a chelator for zirconium-89

Darpan N. Pandya*[1], Pere Miro[2], Michael A. Sinnwell[2,3], George B. Crull[2] and Thaddeus J. Wadas*[1]

[1]Department of Radiology, University of Iowa, Iowa City, Iowa, United States of America; darpan-pandya@uiowa.edu (D.N.P.); Thaddeus-Wadas@uiowa.edu (T.J.W.)
[2]Department of Chemistry, University of Iowa, Iowa City, Iowa, United States of America; Pere-miro@uiowa.edu (P.M.); Michael-sinwell@uiowa.edu (M.A.S.); George-crull@uiowa.edu (G.B.C.)
[3]MATFab Facility, University of Iowa, Iowa City, Iowa, United Sates of America; Michael-sinwell@uiowa.edu (M.A.S.)
* Correspondence: Thaddeus-wadas@uiowa.edu; 1.319.335.5009 (T.J.W.); darpan-pandya@uiowa.edu; (1.319.335.1520. (D.N.P.)

*The corresponding authors for this manuscript are listed below.

Thaddeus J. Wadas, Ph.D.
Associate Professor of Radiology
Director, Small Animal Imaging Core
Carver College of Medicine
University of Iowa
169 Newton Road
Iowa City, IA 52242
phone: (319) 335-5009
fax: (319) 353-6275
e-mail: thaddeus-wadas@uiowa.edu

Darpan N. Pandya, Ph.D.
Associate Research Scientist
Department of Radiology
Carver College of Medicine
University of Iowa
169 Newton Road
Iowa City, IA 52242
phone: (319) 335-5161
e-mail: darpan-pandya@uiowa.edu

Table of Contents

**Section**	**Page Number**
Solid State ^{13}C-NMR of DOTMA and Zr-DOTMA (Figure S1)	3
Solid State ^{15}N-NMR of DOTMA and Zr-DOTMA (Figure S2)	4
Selected geometrical parameters of Zr-DOTA and Zr-DOTMA in gas phase and in solution. Experimental Zr-DOTA geometrical parameters in parenthesis (Table S1)	5
Radiochemistry protocol using $[^{89}Zr]Zr(ox)_4$ for $[^{89}Zr]$Zr-DOTMA (Table S2, Figure S3)	6-7
Preparation of $[^{89}Zr]ZrCl_4$ from $[^{89}Zr]Zr(ox)_4$ (Figure S4)	8
Summary of optimized reaction buffers using $[^{89}Zr]ZrCl_4$ for $[^{89}Zr]$Zr-DOTMA (Table S3, Figure S5)	9-10
Summary of optimized reaction temperatures using $[^{89}Zr]ZrCl_4$ for $[^{89}Zr]$Zr-DOTMA (Table S4, Figure S6)	11-12
Quality control of $[^{89}Zr]$Zr-DOTMA by radio-TLC (Figure S7)	13
Summary of optimized radiochemistry conditions to prepare $[^{89}Zr]$Zr-DOTMA with $[^{89}Zr]ZrCl_4$ (Table S5)	14
Determination of partition coefficients (LogP) (Table S6)	15
In vitro serum stability study by Radio-ITLC (Figure S8, S9)	16-17
Complete biodistribution studies of $[^{89}Zr]$Zr-DOTMA (Table S7)	18

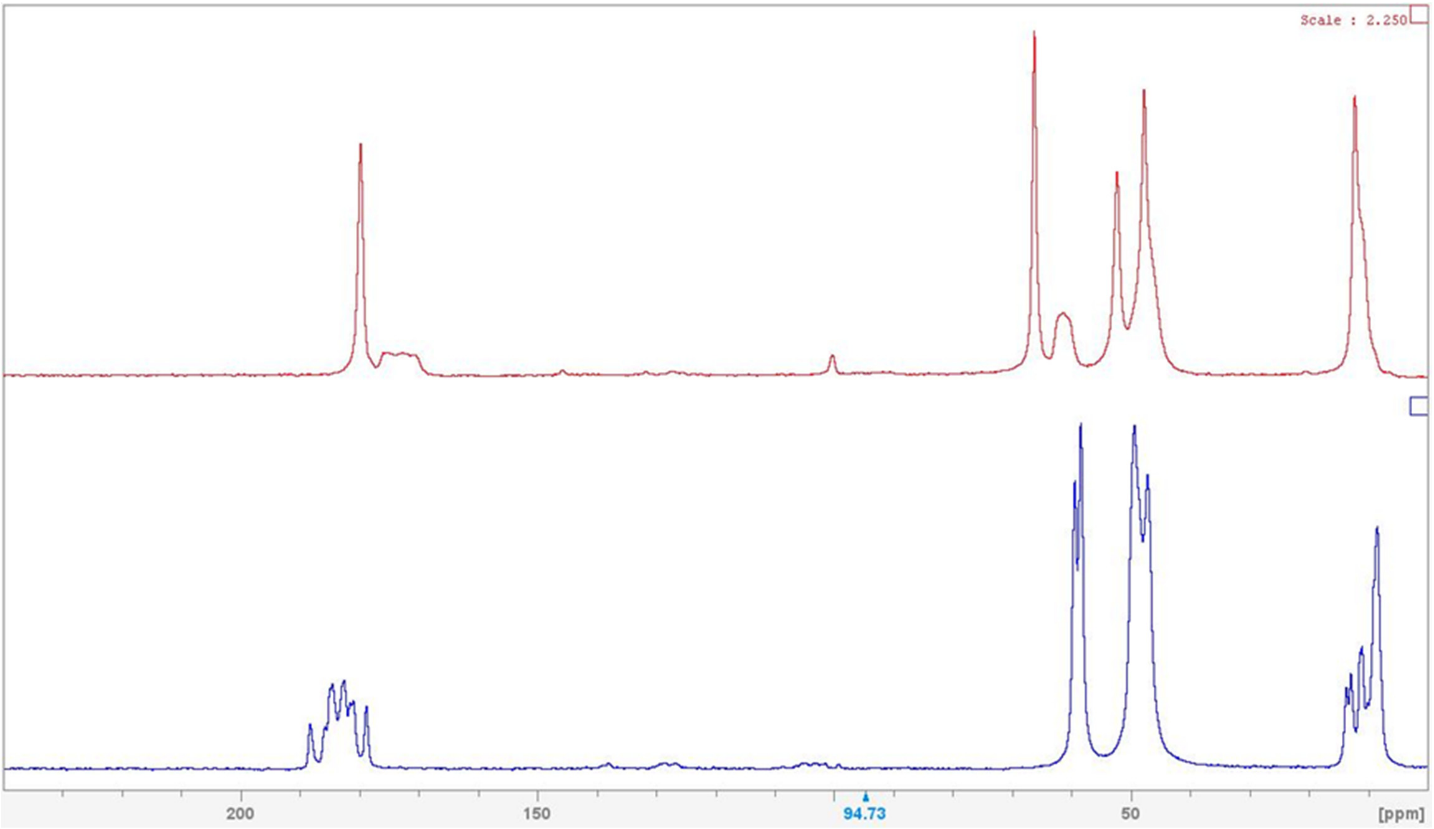

Figure S1: The Carbon CPMAS spectra of DOTMA (Bottom) and Zr-DOTMA (Top). The DOTMA material is consistent with a mixture of crystalline phase. Upon complexation with Zr a crystalline material unique from the free material was observed. The Zr-DOTMA is consistent with a primarily major crystalline phase. The sample also has a minor phase. The Zr-DOTMA is clearly unique from the DOTMA.

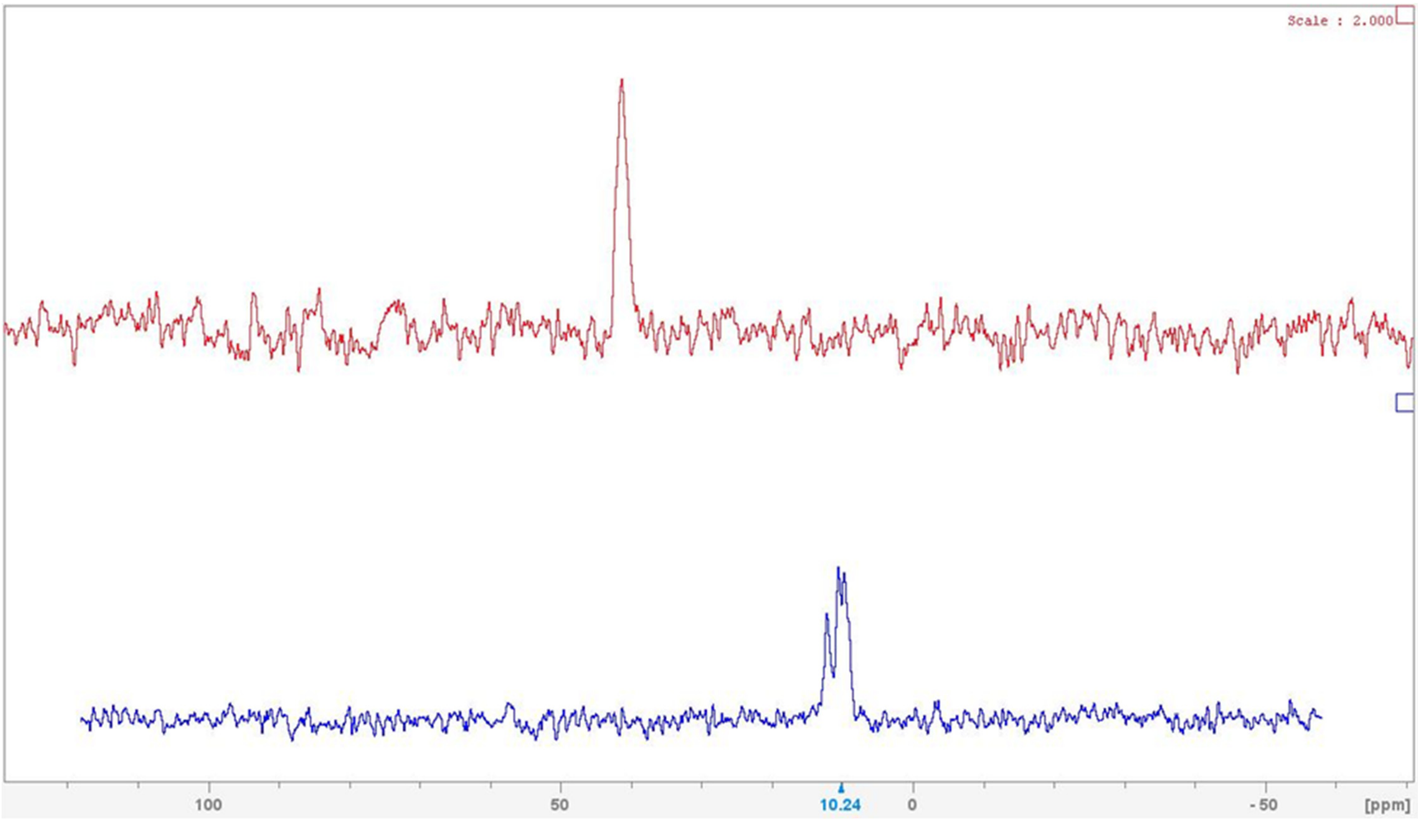

Figure S2: The Nitrogen CPMAS spectra of DOTMA (Bottom) and Zr-DOTMA (Top). The DOTMA material is consistent with nitrogen atoms in at least three unique locations. It is not clear if this is from multiple phases (as suggested by the carbon data) or ring puckering. Upon complexation with Zr a crystalline material unique from the free material was observed. The Zr-DOTMA is consistent with a single crystalline phase which are clearly unique from the DOTMA. The resonance is sharp and suggest a high symmetry environment.

Table S1. Selected geometrical parameters of Zr-DOTA and Zr-DOTMA in gas phase and in solution. Experimental Zr-DOTA geometrical parameters in parenthesis.

	**Zr-DOTA**	**Zr-DOTMA**
Zr-N (Å)	2.486 (2.415)	2.503
Zr-O (Å)	2.138 (2.134)	2.127
N-Zr-N (°)	71.65 (72.87)	72.27
O-Zr-O (°)	78.74 (77.21)	78.30

Table S2. Summary of optimized radiochemistry conditions to prepare [^{89}Zr]Zr-DOTMA using [^{89}Zr]Zr(ox)$_4$.

Radiochemistry conditions	DOTMA (*n* = 10)
Quantity (µg)	50
[^{89}Zr]Zr(ox)$_4$ added (MBq)	16.5-20.5
Reaction Buffer	0.5 M HEPES
Final Reaction pH	6.9-7.2
Reaction Temperature (°C)	99
Reaction time (min)	120
Radiolabeling yield (%)	36 ± 5.1

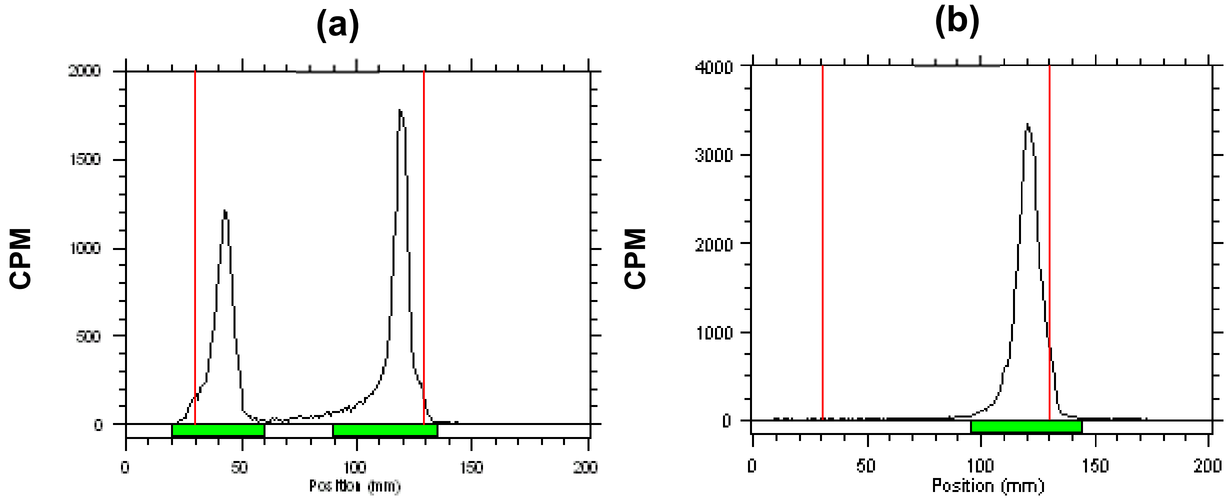

Figure S3. Radio-TLC analysis of [^{89}Zr]Zr-DOTMA prepared by using [^{89}Zr]Zr(ox)$_4$ at 99 °C for 120 min. (a) [^{89}Zr]Zr-DOTMA, (b) [^{89}Zr]Zr(ox)$_4$. In this ITLC-SA system, unchelated ^{89}Zr forms a complex with EDTA and elutes with the solvent front (R_f = 0.9), while [^{89}Zr]Zr-DOTMA complex moves from origin (R_f = 0.10-0.15).

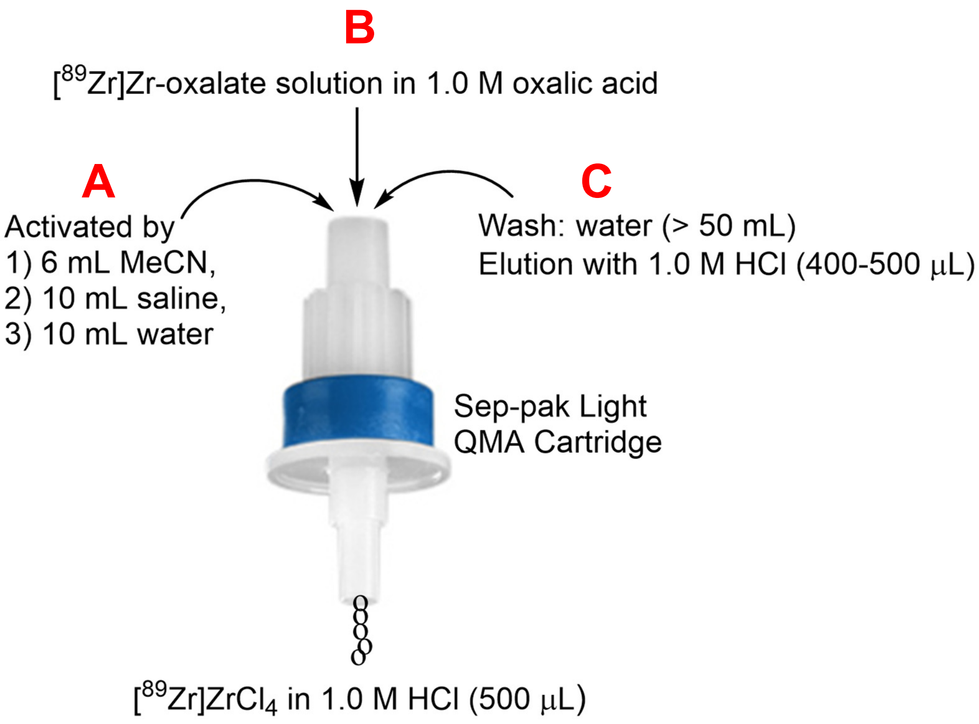

Figure S4. Schematic diagram for the production of [^{89}Zr]ZrCl$_4$ from [^{89}Zr]Zr(ox)$_4$.

Table S3. Summary of optimized radiochemistry conditions and [^{89}Zr]ZrCl$_4$ activity used to prepare [^{89}Zr]Zr-DOTMA[a] complex (*n* = 4 for each buffer)

Quantity of Ligand	[^{89}Zr]ZrCl$_4$ added (MBq)	Reaction Buffer (pH 6.8 - 7.2)	Reaction Temperature (°C)	Reaction Time (min)	Radiochemical Yield by Radio-ITLC (%)
DOTMA (10 μg)	20.1	1 M NH_4OAc	95	60	57.2 ± 1.1
	21.2	1 M NaOAc	95	60	47.9 ± 0.8
	22.3	1 M TRIS	95	60	22.7 ± 1.4
	20.8	1 M TMAA	95	60	66.5 ± 0.9
	23.5	0.5 M MES	95	60	89.9 ± 0.5
	**24.0**	**0.5 M HEPES**	**95**	**60**	**99.9 ± 0.1**

[a]DOTMA ligand (10 μg) was labeled with [^{89}Zr]ZrCl$_4$ (20.1 – 24.0 MBq) using buffers (200 μL, pH 6.8 - 7.2) at 95 °C for 60 min. NH_4OAc: Ammonium acetate; NaOAc: Sodium acetate; TMAA: Tetramethylammonium acetate; MES: 2-(N-morpholino)ethanesulfonic acid; HEPES: 4-(2-Hydroxyethyl) piperazine-1-ethanesulfonic acid; TRIS: Tris(hydroxymethyl)aminomethane.

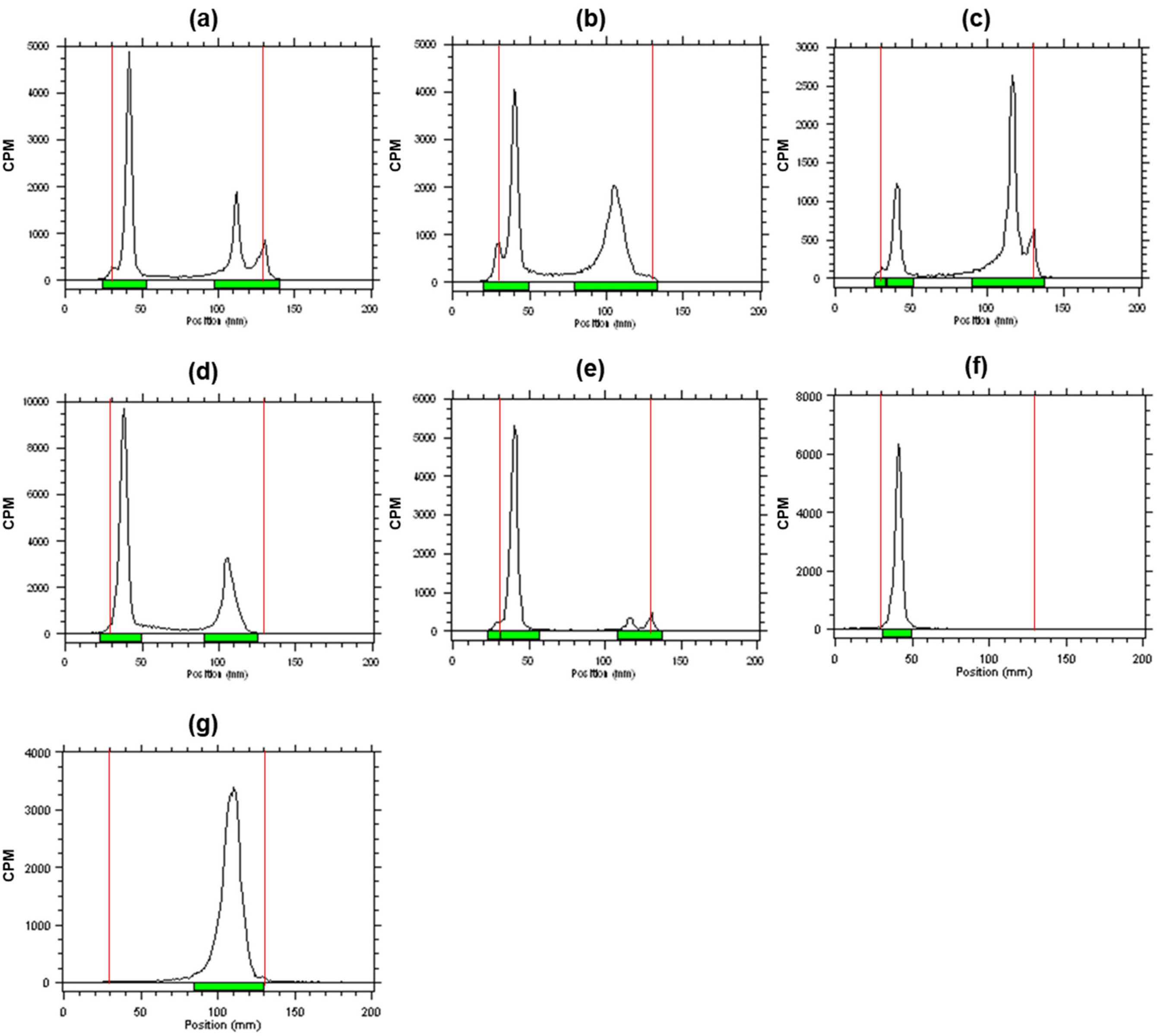

Figure S5. Radio-TLC analysis of [^{89}Zr]Zr-DOTMA prepared by using different buffers at 99 °C for 60 min. (a) 1 M NH_4OAc, (b) 1 M NaOAc, (c) 1 M TRIS, (d) 1 M TMAA, (e) 0.5 M MES, (f) 0.5 M HEPES, (g) [^{89}Zr]$ZrCl_4$. In this ITLC-SA system, free ^{89}Zr forms a complex with EDTA and elutes with the solvent front (R_f ~ 0.9), while [^{89}Zr]Zr-DOTMA complex moves from origin (R_f = 0.10-0.15).

Table S4. Summary of optimized reaction temperature and [^{89}Zr]$ZrCl_4$ activity used to prepare [^{89}Zr]Zr-DOTMA[a] complex (*n* = 4 for each temperature)

Quantity of Ligand	[^{89}Zr]$ZrCl_4$ added (MBq)	Reaction Buffer (pH 6.8 - 7.2)	Reaction Temperature (°C)	Reaction Time (min)	Radiochemical Yield by Radio-ITLC (%)
DOTMA (10 μg)	20.0	0.5 M HEPES	40	60	24.8 ± 1.3
	20.8	0.5 M HEPES	50	60	35.3 ± 0.7
	21.5	0.5 M HEPES	60	60	58.3 ± 1.5
	20.9	0.5 M HEPES	70	60	81.9 ± 0.5
	22.5	0.5 M HEPES	80	60	93.1 ± 0.4
	**23.0**	**0.5 M HEPES**	**95**	**60**	**99.9 ± 0.1**

[a]DOTMA ligand (10 μg) was labeled with [^{89}Zr]$ZrCl_4$ (20.0 – 23.0 MBq) using 0.5 M HEPES buffer (200 μL, pH 6.8 - 7.2) at different temperatures (40 - 95 °C) for 60 min.

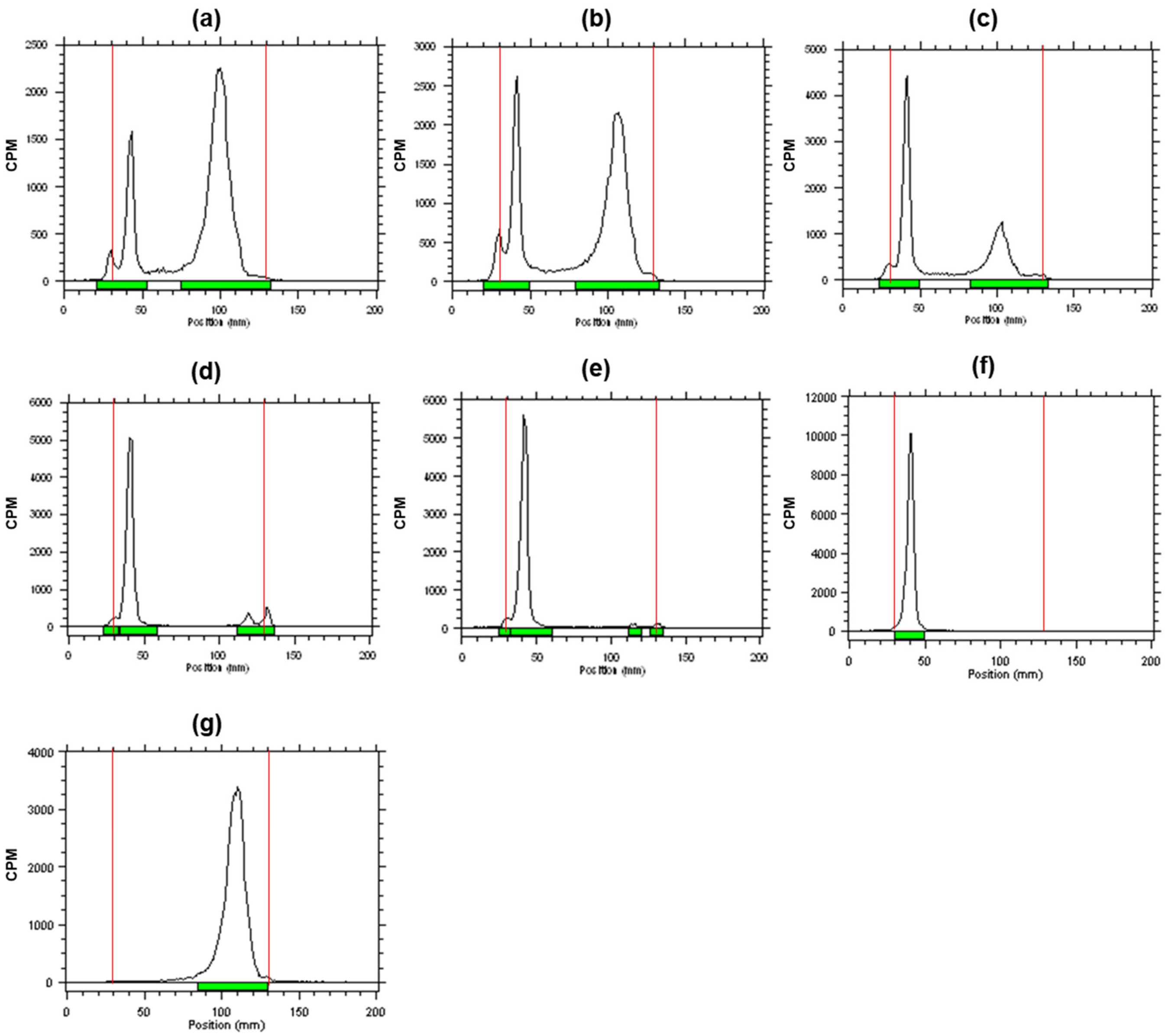

Figure S6. Radio-TLC analysis of [^{89}Zr]Zr-DOTMA prepared by using 0.5 M HEPES buffer (pH 6.8 - 7.2) at different temperatures (40 - 95 °C) for 60 min. (a) 40 °C, (b) 50 °C, (c) 60 °C, (d) 70 °C, (e) 80 °C, (f) 95 °C, (g) [^{89}Zr]$ZrCl_4$. In this ITLC-SA system, free ^{89}Zr forms a complex with EDTA and elutes with the solvent front (R_f ~ 0.9), while [^{89}Zr]Zr-DOTMA complex moves from origin (R_f = 0.10-0.15).

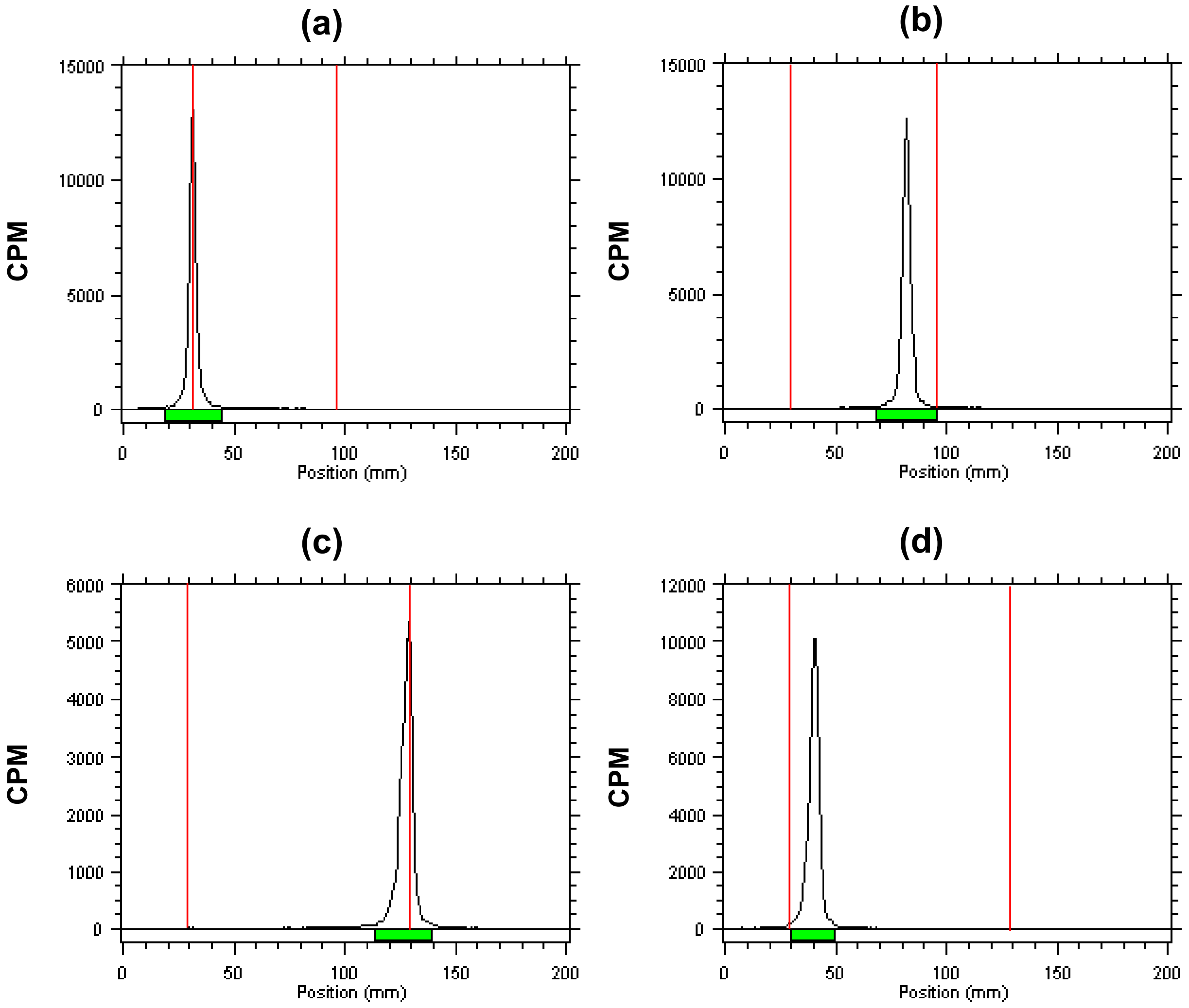

Figure S7. Quality control of [^{89}Zr]Zr-DOTMA by radio-TLC. C-18-TLC of [^{89}Zr]$ZrCl_4$ (a), [^{89}Zr]Zr-DOTMA (b), and ITLC-SA of [^{89}Zr]$ZrCl_4$ (c), [^{89}Zr]Zr-DOTMA (d)

Table S5. Summary of optimized radiochemistry conditions to prepare [^{89}Zr]Zr-DOTMA using [^{89}Zr]ZrCl$_4$.

**Radiochemistry conditions**	**DOTMA (*n* = 30)**
Quantity (μg)	15-20
[^{89}Zr]ZrCl$_4$ added (MBq)	40.7-59.2
Reaction Buffer	0.5 M HEPES
Final Reaction pH	6.9-7.2
Reaction Temperature (°C)	95
Reaction time (min)	60
Radiochemical yield (%)	≥ 99.99
Molar activity (A_m; MBq μmol^{-1})	1055 ± 6

Table S6. LogP value for [^{89}Zr]Zr-DOTMA

Complex	LogP (*n*=5)
[^{89}Zr]Zr-DOTMA	-2.97 ± 0.02

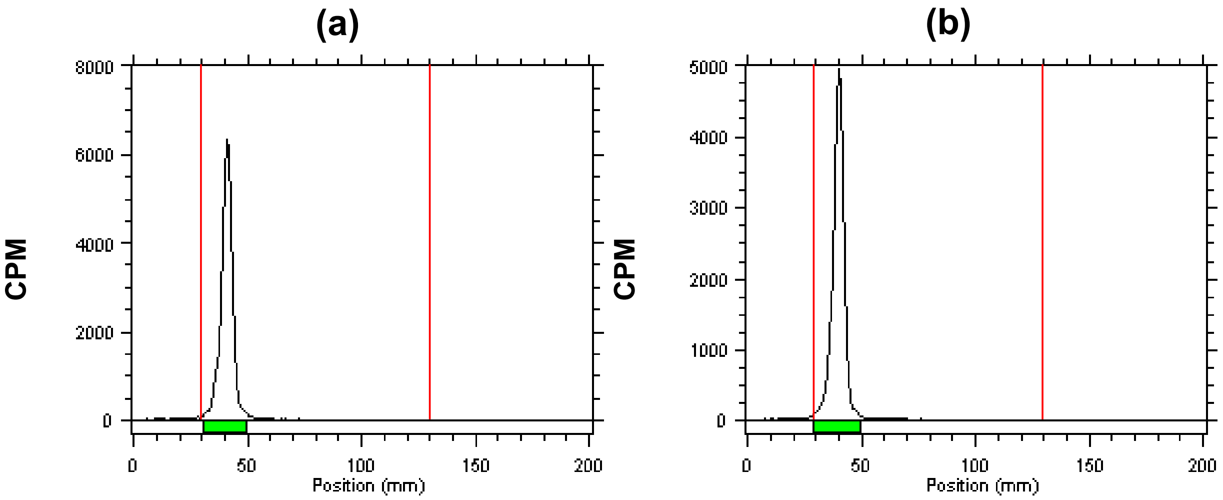

Figure S8. Radio-ITLC of [^{89}Zr]Zr-DOTMA solution in human serum at 37 °C after 0 h (a) and 7 days (b).

When samples of serum which contained unchelated [^{89}Zr]Zr-DOTMA were analyzed using radio-ITLC, the radiometal comlex remained at the origin.

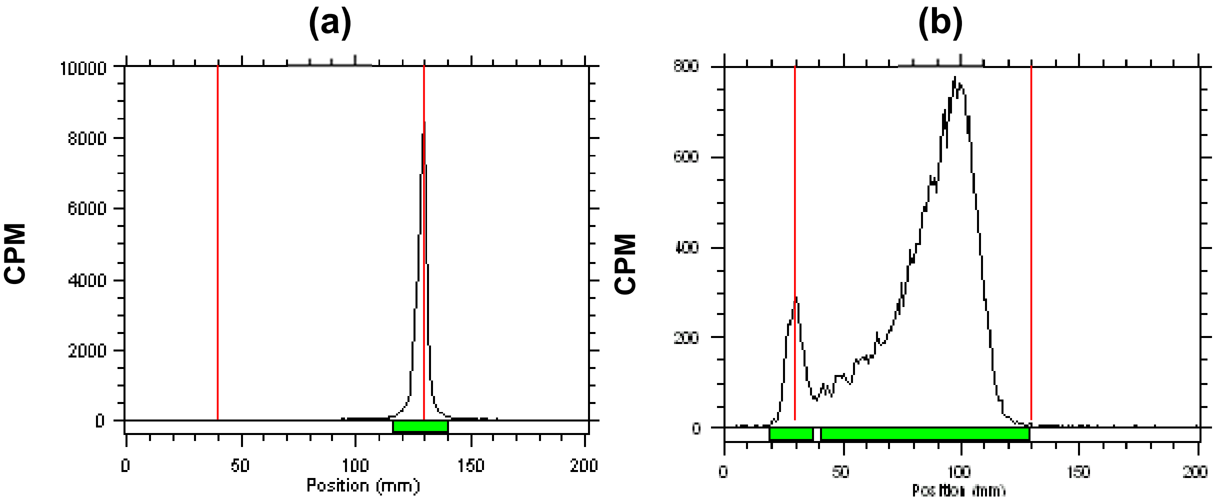

Figure S9. Radio-ITLC of [^{89}Zr]$ZrCl_4$ solution in human serum at 37 °C after 0 h (a) and 7 days (b). When samples of serum which contained unchelated [^{89}Zr]$ZrCl_4$ were analyzed using radio-ITLC, we observed broad peaks ranging from the origin to the solvent front because unchelated ^{89}Zr could be bound to variety of serum protein components and migrate with different retention factors in our ITLC system. Therefore, we elected to perform size exclusion chromatography to further characterize the stability of ^{89}Zr-complexes.

Table S7. Biodistribution (%ID/g) of [^{89}Zr]Zr-DOTMA in selected organs at 2, 4, 24, 48, and 72 h p.i. (*n* = 6/timepoint)

Tissue/Organ	2 h	4 h	24 h	48 h	72 h
Blood	0.011 ± 0.001	0.008 ± 0.001	0.005 ± 0.001	0.004 ± 0.001	0.003 ± 0.001
Heart	0.021 ± 0.003	0.019 ± 0.004	0.009 ± 0.002	0.004 ± 0.002	0.004 ± 0.001
Lung	0.071 ± 0.006	0.060 ± 0.005	0.033 ± 0.004	0.015 ± 0.002	0.013 ± 0.003
Liver	0.238 ± 0.020	0.123 ± 0.017	0.018 ± 0.002	0.008 ± 0.001	0.007 ± 0.001
Small intestine	0.229 ± 0.039	0.080 ± 0.007	0.007 ± 0.001	0.002 ± 0.000	0.001 ± 0.000
Large intestine	0.523 ± 0.112	0.632 ± 0.088	0.022 ± 0.008	0.003 ± 0.001	0.003 ± 0.001
Kidney	1.053 ± 0.071	0.689 ± 0.086	0.077 ± 0.009	0.021 ± 0.003	0.010 ± 0.002
Spleen	0.078 ± 0.008	0.076 ± 0.004	0.044 ± 0.005	0.024 ± 0.005	0.026 ± 0.002
Pancreas	0.011 ± 0.003	0.010 ± 0.004	0.003 ± 0.001	0.001 ± 0.001	0.001 ± 0.001
Stomach	0.069 ± 0.008	0.013 ± 0.005	0.005 ± 0.002	0.001 ± 0.000	0.001 ± 0.000
Muscle	0.008 ± 0.003	0.004 ± 0.002	0.001 ± 0.000	0.001 ± 0.001	0.001 ± 0.001
Fat	0.007 ± 0.003	0.006 ± 0.002	0.005 ± 0.002	0.002 ± 0.001	0.001 ± 0.001
Bone	0.032 ± 0.006	0.024 ± 0.004	0.026 ± 0.004	0.023 ± 0.004	0.023 ± 0.003
